# Analysis of Wavelet Coherence in Calf Agonist-Antagonist Muscles during Dynamic Fatigue

**DOI:** 10.3390/life14091137

**Published:** 2024-09-09

**Authors:** Xindi Ni, Loi Ieong, Mai Xiang, Ye Liu

**Affiliations:** School of Sport Science, Beijing Sport University, Beijing 100084, China; nxd_bsu@foxmail.com (X.N.); loi_326@hotmail.com (L.I.); michael9789@126.com (M.X.)

**Keywords:** muscle fatigue, wavelet coherence, antagonistic muscles, EMG

## Abstract

Dynamic muscle fatigue during repetitive movements can lead to changes in communication between the central nervous system and peripheral muscles. This study investigated these changes by examining electromyogram (EMG) characteristics from agonist and antagonist muscles during a fatiguing task. Twenty-two healthy male university students (age: 22.92 ± 2.19 years) performed heel raises until fatigue. EMG signals from lateral gastrocnemius (GL) and tibialis anterior (TA) muscles were processed using synchrosqueezed wavelet transform (SST). Root mean square (RMS), mean frequency (MF), power across frequency ranges, wavelet coherence, and co-activation ratio were computed. During the initial 80% of the task, RMS and EMG power increased for both muscles, while MF declined. In the final 20%, GL parameters stabilized, but TA showed significant decreases. Beta and gamma intermuscular coherence increased upon reaching 60% of the task. Alpha coherence and co-activation ratio remained constant. Results suggest that the central nervous system adopts a differentiated control strategy for agonist and antagonist muscles during fatigue progression. Initially, a coordinated “common drive” mechanism enhances both muscle groups’ activity. Later, despite continued increases in muscle activity, neural-muscular coupling remains stable. This asynchronous, differentiated control mechanism enhances our understanding of neuromuscular adaptations during fatigue, potentially contributing to the development of more targeted fatigue assessment and management strategies.

## 1. Introduction

During human motion, the central nervous system regulates the active and antagonist muscles’ cooperation through feed-forward adjustments via descending signals and feedback adjustments from ascending signals, enabling the execution of various complex movements [1]. The modulatory role of descending signals commonly manifests as oscillations in the beta and gamma frequency bands in EMG signals, often interpreted using De Luca’s “common drive” concept [2,3]. Meanwhile, ascending feedback regulatory information typically appears as oscillations in the alpha frequency band [4,5]. The weakening of bidirectional signal communication between the cerebral cortex and muscles is thought to be the primary neurophysiological mechanism driving muscle fatigue [3]. However, this perspective largely stems from studies comparing non-fatigued and fatigued states [3,6], potentially overlooking the nuances of fatigue evolution [7].

Fatigue resulting from prolonged physical activity is a progressive process [7]. During fatigue onset, an increase in the discharge rate of motor unit action potentials (MUAP) is observed, followed by the accumulation of fatigue [8]. Concurrently, a decline in local muscle conduction velocity during fatigue leads to a reduction in muscle EMG amplitude [9]. Moreover, fatigue can also affect antagonist muscle EMG activity and co-activation levels [6,10,11,12]. Research has observed the H-reflex of the antagonist-prime mover pair in the calf during isometric fatigue processes, identifying a phenomenon of bidirectional spinal reflex excitability regulation in the antagonist muscle during fatigue [13,14]. This regulation of antagonist muscles seems to be modulated by structures of the spine, aiming to maintain a specific functional level [12]. However, when this modulation occurs, and what its relationship with prime mover and antagonist muscle activity is, remains unclear. Hence, further understanding of the neuromuscular system’s changes during the progression of fatigue is crucial for comprehending fatigue [7,15].

The central nervous system drives muscle movement by regulating the discharge frequency of muscles. These different frequency muscle electrical activities are often considered manifestations of functional activities in different brain circuits [16]. Such manifestations are commonly studied through EEG-EMG coherence or EMG-EMG coherence [11,17,18,19,20]. Research has suggested that EMG alpha band coherence may originate from the reticular spinal cord [18], possibly reflecting ascending or feedback interactions and being influenced by Renshaw cells in the spinal cord [5]. The beta and gamma bands are primarily driven by the motor cortex [21]. Previous studies have found strong beta frequency band coherence during postural tasks, while gamma frequency band (31–60 Hz) coherence is associated with dynamic force output [22]. These results indicate that different tasks induce changes in cortico-muscular coherence of different frequency bands. Coherence research has mainly focused on long-term recordings of basic, quasi-static physiological activities [1,16], consistently observing an increase in EMG beta coherence [5,11]. Dynamic movements have been observed to shift beta band coherence to the gamma band [1]. As most human dynamic movements involve both isometric and isotonic contractions, studying the changes in coherence and co-activation of different frequency bands during the dynamic fatigue process can more comprehensively reflect the central nervous system’s regulation of muscles.

Although intermuscular coherence analysis has been widely used to reflect the neuromuscular control mechanism of movement, most related research uses the Fourier coherence method. This power spectrum estimation method, based on periodograms, has limitations. For instance, spectral leakage after windowing and large variance may affect the reliability of results [23]. Furthermore, in most movements and some pathological conditions, the actions are continuous, making the Fourier coherence method potentially unsuitable [16]. This necessitates the use of non-stationary analysis methods, such as wavelet methods. Using wavelet methods for coherence analysis can not only improve the accuracy of coherence but also accommodate non-stationary signals [1]. Wavelet transform is a common and mature method that can address the non-stationarity, randomness, and multi-component properties of sEMG signals. It relies on the mother wavelet to represent signals in the frequency space and offers higher accuracy than traditional methods [24]. The Synchrosqueezing Wavelet Transform (SST), developed from the Continuous Wavelet Transform (CWT), provides better frequency resolution. Moreover, SST has shorter computation time and better stability against errors [25]. Therefore, using SST, we can perform coherence analysis with high time-frequency resolution.

The aim of this study is to use the SST method, which is more suitable for dynamic signals, to investigate the changes in intermuscular coherence and co-activation in the alpha, beta, and gamma frequency bands of the prime mover and antagonist muscles during the fatigue process. As calf muscles play a crucial role in both daily activities and sports performance [26], the gastrocnemius muscle’s high proportion of Type I fibers [27] leads to a gradual fatigue process, offering a better opportunity to study neuromuscular changes. By using the calf as an example, we aim to understand the changes in neuromuscular control during the dynamic fatigue process in calf muscles.

## 2. Materials and Methods

### 2.1. Subjects

A total of 22 healthy participants were recruited from the student population of Beijing Sport University (all males; age: 22.92 ± 2.19 years, average height: 1.79 ± 0.06 m, average weight: 76.08 ± 9.01 kg; all participants were right-leg dominant). Participants were recruited through convenience sampling from the student population of Beijing Sport University. The study was approved by the Ethics Committee of Beijing Sport University, and all participants received an informed consent form that they signed prior to the experiment. The EMG of the gastrocnemius and tibialis anterior of each participant’s dominant leg was analyzed. Inclusion criteria: males aged between 18 and 25 years old, in good health (defined as having no history of cardiovascular, respiratory, musculoskeletal, or neurological disorders), no history of lower limb injuries in the past one year, and not systematically trained athletes or sports students. Exclusion criteria: fear of the experiment; allergy to any part of the instruments used in the measurement; presence of infections or signs of fatigue on the assessment day; consumption of medications, drugs (including antidepressants and pain medications), or caffeine on the assessment day that may influence neuromuscular function during the fatigue test.

### 2.2. Experimental Protocol

All measurements were conducted in the Biomechanics Laboratory of Beijing Sport University. The recordings were carried out on a 30 cm high jump box. The EMG of the GL and TA of the dominant leg of the participants was measured. The fatigue protocol included performing heel raises at a rhythm of 75 BPM. Participants were allowed to put their fingers on a wall to maintain balance and were instructed to keep their knees straight and try to achieve the maximum amplitude during heel raise. EMG signals were continuously recorded throughout the experiment, and the Rating of Perceived Exertion (RPE) was recorded every 30 s. The experiment was terminated when the participant’s RPE ≥ 18 and they could not follow the specified rhythm. The RPE used the Borg Scale [28], ranging from 6 (very, very light) to 20 (very, very hard). The employed calf fatigue protocol is a commonly used test for assessing calf muscle function under clinical conditions. It can test the endurance of the calf muscles, and in this test, both the RMS amplitude and MF of the EMG signal change progressively during repeated heel raises [29]. Subjects were verbally motivated to give their best effort.

The Delsys wireless EMG system was used to record muscle activity. It is an amplifier with a gain of 1000. It was set to record at a sampling frequency of 1926 Hz. The EMG system included four 5 × 1 mm^2^ Ag electrodes with an inter-electrode spacing of 10 mm, arranged in a 2 × 2 configuration. Skin preparation included shaving and wiping the skin with alcohol. Electrode placements followed the recommendations of the ISEK tutorials (Merletti, R., and G. L. Cerone, 2020) [30]. Electrodes were placed in the middle of the belly of the GL and TA muscles, parallel to the direction of the muscle fibers. The EMG acquisition module was bound with an elastic band to minimize the occurrence of motion artifacts.

### 2.3. Data Processing

Data were analyzed off-line using custom-written programs with MATLAB 2022b (The MathWorks Inc., Natick, MA, USA) to apply all necessary processing to obtain spectral representations of the EMG signals. First, each participant’s signal was normalized using the maximum value of the signal during the entire fatigue process. This was followed by preprocessing with a 4th order Butterworth band-pass filter (5–500 Hz) to reduce motion artifacts and high-frequency noise. Research has shown [7] that dividing signals into 10 segments during wavelet coherence analysis can reveal details of fatigue effectively. Thus, in this study, each participant’s signal was divided into 10 equal segments according to its duration for subsequent analysis.

### 2.4. Synchrosqueezed Wavelet Transform

In order to extract the power of EMG signals at different frequency bands, we first used the SST method to calculate the time-frequency matrix of the segmented EMG signals. SST, based on the Continuous Wavelet Transform (CWT), reassigns the signal to improve the time-frequency (TF) representation.

In this study, we chose the Morlet wavelet as the mother wavelet due to its excellent frequency localization characteristics. The Morlet wavelet is defined as follows:

(1) $$\psi (t)=\frac{1}{\sqrt[{4}]{\pi }}(e^{i\omega_{0}t}-e^{\frac{-\omega_{0}^{2}}{2}})e^{-\frac{t^{2}}{2}}$$

where *ψ(t)* represents the Morlet wavelet, *t* is the non-dimensional time, *i* is the imaginary unit, *ω_0_* is the non-dimensional frequency of the Morlet wavelet, and *e* is the base of the natural logarithm. The surface electromyographic signal *e(t)* is first decomposed into a continuous wavelet representation *We(a, b)*, defined as follows:

(2) $$W_{e}(a,b)=\frac{1}{\sqrt{a}}\int_{-\infty }^{\infty }e(t)\psi^{*}(\frac{t-b}{a})dt$$

Here, *a* represents scale, and *b* represents time shift. When choosing the mother wavelet *ψ*, two conditions need to be satisfied: (i) the Fourier transform of the wavelet has strictly positive support; (ii) the wavelet satisfies the standard admissibility condition, as shown in the following formula:

(3) ∫0∞z−1ψ^(z)dz<∞

Next, we calculate the derivative of the complex phase of *We(a, b)* to obtain the phase transform. For any *(a, b)* values satisfying *We(a, b)* ≠ 0, the instantaneous frequency *ωe(a, b)* of the TF spectrum is calculated as follows:

(4) $$\omega_{e}(a,b)=-i{\left(W_{e}(a,b)\right)}^{-1}\frac{\partial }{\partial b}W_{e}(a,b)$$

Afterwards, we map the existing time-scale plane to the TF plane, transforming from *(b, a)* to (b, $\omega_{e}$*(a, b)*). On the TF plane, we estimate the SST, denoted as TS*(ω, b)*, only at the center of the continuous interval *ω*_1_, as shown in the following formula:

(5) $$[\omega_{l}-\frac{1}{2}\Delta \omega ,\omega_{l}+\frac{1}{2}\Delta \omega ]\;with\;\omega_{l}-\omega_{l-1}=\Delta \omega$$

(6) $$T_{s}\left(\omega_{l},b\right)={\left(\Delta \omega \right)}^{-1}\sum_{a_{k}:\left|\omega \left(a_{k},b\right)-\omega_{l}\right|\leq \Delta \omega /2}W_{e}\left(a_{k},b\right)a_{k}^{\frac{-3}{2}}\left(\Delta a\right)k$$

Based on the obtained time-frequency distribution matrix TS, the time-domain integration is performed on different frequency bands in the electromyographic signal to calculate their average power. We will focus on the following frequency bands: alpha, beta, gamma, and high frequency bands.

### 2.5. EMG-EMG Coherence

To obtain the EMG-EMG coherence in different frequency bands, we first calculate the coherence matrix. The coherence matrix is computed using the analytic Morlet wavelet. The wavelet coherence of two time series *x* and *y* is defined as follows:

(7) $$WCoh=\frac{{\left|S(T_{x}^{*}(a,b)T_{y}(a,b))\right|}^{2}}{S({\left|T_{x}(a,b)\right|}_{2})\times S({\left|T_{y}(a,b)\right|}_{2})}$$

Here, *Tx (a, b)* and *Ty(a, b)* denote the SST of *x* and *y* at scales a and positions *b*. The superscript * indicates the complex conjugate and *S* is a smoothing operator in time and scale [31].

### 2.6. RMS, MF and Coactivation Ratio

Root Mean Square (RMS):

(8) $$RMS=\sqrt{\frac{1}{N}\sum_{i=1}^{N}x_{i}^{2}}$$

Median frequency (MF):

(9) $$MF=f_{K}$$

(10) $$\sum_{m=1}^{K}\Phi (f_{m})=\sum_{m=K+1}^{M}\Phi (f_{m})$$

The co-activation index is calculated using the following formula:

(11) $$CI=2*\frac{EMG_{ANT}}{EMG_{AG}+EMG_{ANT}}*100$$

where *EMG_ANT_* represents the EMG of the antagonist muscle, and *EMG_AG_* denotes the EMG of the agonist muscle.

### 2.7. Statistical Analysis

In this study, statistical analysis was carried out using IBM SPSS Statistics 25 software. Data for each individual, divided into ten parts based on time, was subjected to a normality test for the extracted indicators, including different frequency band EMG power, co-activation ratio, first principal component, and intermuscular coherence in different frequency bands. For data not adhering to a normal distribution, a log10 transformation was applied. Subsequently, a one-way repeated measures analysis of variance (ANOVA) was performed.

Prior to conducting the ANOVA, the Mauchly’s sphericity test was initially conducted to assess if the data satisfied the assumption of sphericity. If the assumption was violated, we employed the Greenhouse-Geisser correction method to handle the data, ensuring the accuracy of the results.

Upon identifying statistical significance, post hoc testing was conducted using the LSD method, further comparing differences between time points. The level of significance was set at *p* < 0.05, implying statistical significance when the *p*-value was less than 0.05.

In reporting the experimental results, the data in the text was presented as mean ± standard deviation, while data in graphs was presented as mean ± standard error.

## 3. Results

**Figure 1:**
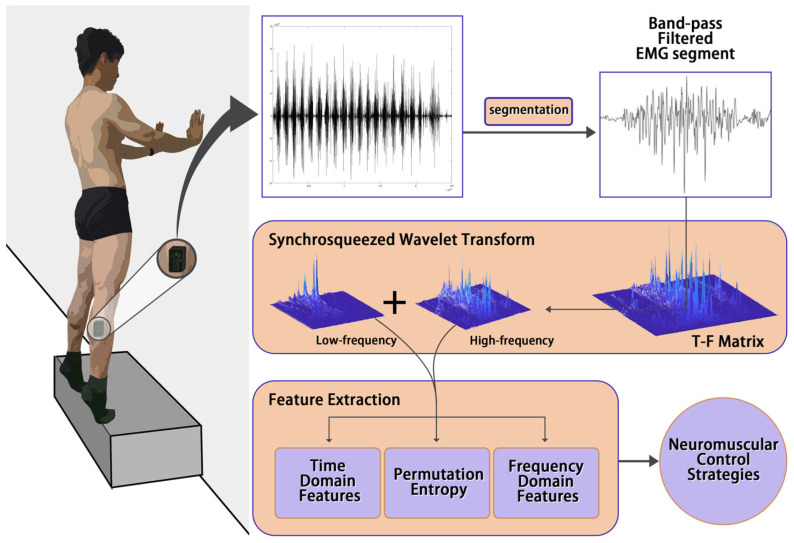
Workflow.

The experimental process is illustrated in Figure 1. During the dynamic contraction phase, the subjects exhibited an average duration of (110.76 ± 58.74) s.

### 3.1. Gastrocnemius Lateralis (GL)

**Figure 2:**
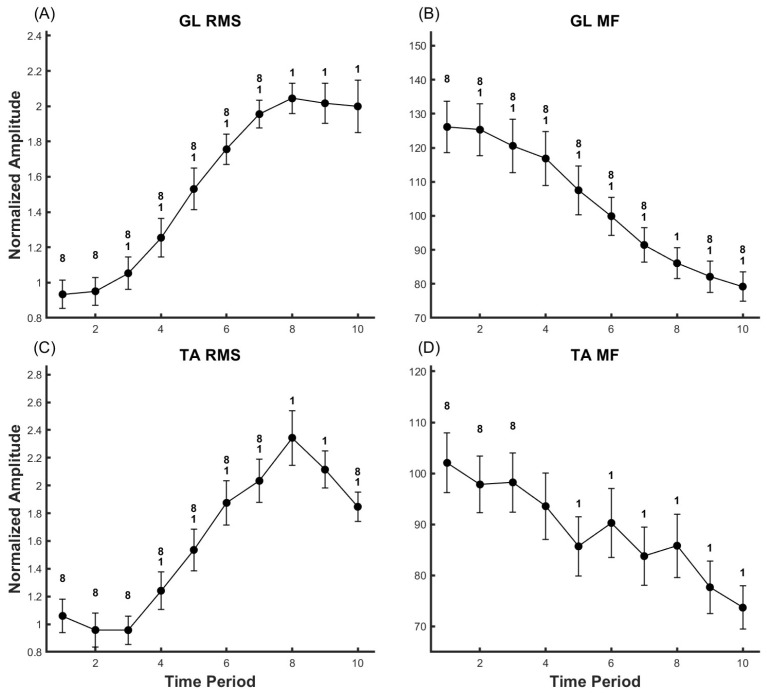
Representing the RMS (**A**,**C**) and MF (**B**,**D**) of EMG for the subjects’ GL and TA muscles. Data are presented as mean ± standard error (SE). ‘1’ indicates significance compared to time point 1 (*p* < 0.05), ‘8’ indicates significance compared to time point 8 (*p* < 0.05).

**Figure 3:**
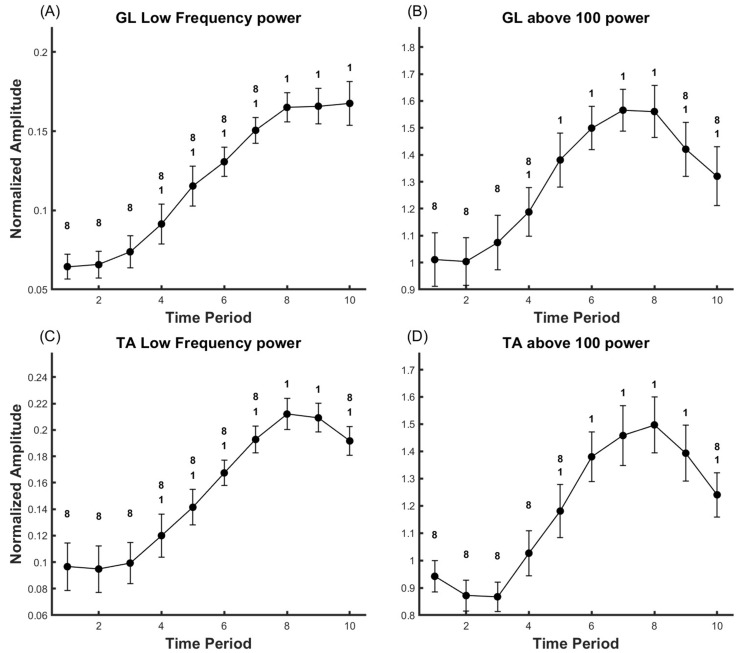
Representing the power changes in low frequency (**A**,**C**) and high frequency (**B**,**D**) of the EMG for GL and TA. Data are presented as mean ± standard error (SE). ‘1’ indicates significance compared to time point 1 (*p* < 0.05), ‘8’ indicates significance compared to time point 8 (*p* < 0.05).

In the GL analysis, as demonstrated in Figure 2 and Figure 3, clear alterations were observed in RMS (Partial Eta Squared = 0.605), MF (Partial Eta Squared = 0.719), the power within the 8–60 Hz frequency band (Partial Eta Squared = 0.691), and the power within the 100–400 Hz frequency band (Partial Eta Squared = 0.321), all of which corresponded with the level of fatigue (*p* < 0.05). Upon post hoc analysis, we discerned the following patterns:

(a) RMS: Compared to the onset of the motion, a significant increase occurred at time points T3 through T10 (*p* < 0.05). However, there were no significant changes noted when comparing T9 and T10 to T8.
(b) MF: There was a significant decline at all points from T2 to T10 when compared to the beginning of the action (*p* < 0.05). In comparison to T8, T9 and T10 also exhibited a significant decrease (*p* < 0.05).
(c) Low frequency band (8–60 Hz): Power significantly increased at time points T4 to T10, compared to the beginning of the action (*p* < 0.05). No significant power changes were observed in T9 and T10 relative to T8 (*p* < 0.05).
(d) Frequency band above 100 Hz: Compared to the onset of the motion, power significantly increased at time points T5 through T10 (*p* < 0.05), while it significantly decreased in T9 and T10 when compared to T8 (*p* < 0.05).

### 3.2. Tibialis Anterior (TA)

In the TA analysis, as shown in Figure 2 and Figure 3, significant changes were observed in RMS (Partial Eta Squared = 0.532), MF (Partial Eta Squared = 0.392), the power within the 8–60 Hz frequency band (Partial Eta Squared = 0.384), and the power within the 100–400 Hz frequency band (Partial Eta Squared = 0.331) in response to increasing fatigue levels (*p* < 0.05). Following post hoc analysis, we identified the following trends:

(a) RMS: Compared to the onset of the motion, there was a significant increase at time points T4 through T10 (*p* < 0.05). Nevertheless, a significant decrease was observed at T10 when compared to T8.
(b) MF: A significant decline was observed at all time points from T5 through T10 when compared to the beginning of the action (*p* < 0.05). However, there were no significant changes noted when comparing T9 and T10 to T8.
(c) Low frequency band (8–60 Hz): Compared to the onset of the motion, power significantly increased at time points T4 through T10 (*p* < 0.05). However, significant power decrease was observed at T10 when compared to T8 (*p* < 0.05).
(d) Frequency band above 100 Hz: Compared to the onset of the motion, power significantly increased at time points T5 through T10 (*p* < 0.05), while it significantly decreased at T10 when compared to T8 (*p* < 0.05).

### 3.3. Co-Activation and EMG-EMG Coherence

**Figure 4:**
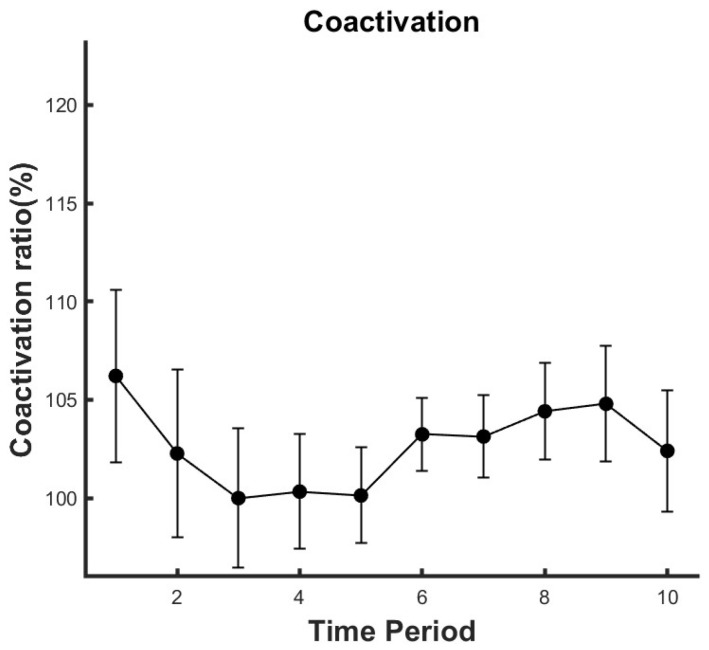
The co-activation changes between GL and TA muscles. Data are presented as mean ± standard error (SE).

Throughout the entire fatigue process, no significant changes were observed in the co-activation ratio (Partial Eta Squared = 0.031) as the level of fatigue deepened (*p* > 0.05), as shown in Figure 4.

**Figure 5:**
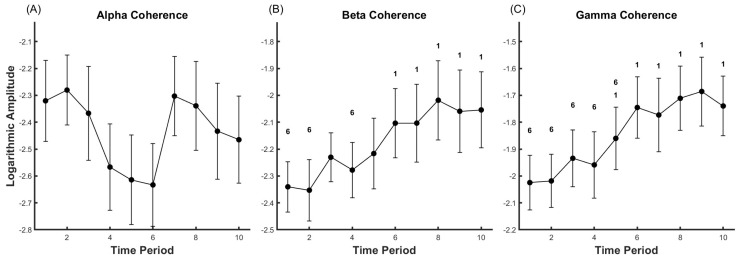
Representing the coherence of alpha (**A**), beta (**B**), and gamma (**C**) in the EMG of GL and TA. Data are presented as mean ± standard error (SE). ‘1’ indicates significance compared to time point 1 (*p* < 0.05), ‘6’ indicates significance compared to time point 6 (*p* < 0.05).

During the fatigue process, significant changes were observed in the beta band coherence (Partial Eta Squared = 0.192) and gamma band coherence (Partial Eta Squared = 0.277) of GL and TA with the intensification of fatigue (*p* < 0.05). However, the alpha band coherence (Partial Eta Squared = 0.047) did not exhibit significant changes with the increase in fatigue (*p* > 0.05), as depicted in Figure 5.

Upon post hoc analysis, we made the following observations.

In the beta frequency band: Compared to the onset of the motion, there was a significant increase from T6 to T10 (*p* < 0.05). Significant differences were observed when comparing T1, T2, and T4 to T6.

In the gamma frequency band: There was a significant increase from T5 to T10 when compared to the beginning of the action (*p* < 0.05). Significant differences were noted when comparing T1 to T5 with T6.

## 4. Discussion

Our study reveals a consistent decline in the MF of the GL muscle during fatigue tasks, aligning with previous findings in fatigue research [11,13,32,33]. We also observed a gradual increase in the RMS of the EMG signals from GL, accompanied by a sustained drop in MF. According to the Joint Analysis of EMG Spectrum and Amplitude (JASA) method [34], these results suggest the successful induction of fatigue in the GL.

The primary findings of this study underscore distinct variations in the RMS and power of the low-frequency band of the GL and TA at the T10 stage during fatigue contractions. Notably, significant increases in the beta and gamma intermuscular coherence of TA and GL were detected at the midpoint of the fatigue task (T6). However, no significant alterations were observed in the alpha intermuscular coherence and co-activation ratio throughout the task.

### 4.1. EMG Time Domain Changes in the Agonist GL and Antagonist TA Muscles

According to Henneman’s size principle [35], MUs are recruited in an orderly manner, from those exerting the weakest force to those generating the most substantial force. When MUs are enlisted, there is a sudden increase in the discharge rate, resulting in an enhancement of EMG power. Our results indicate a significant increase in the RMS of the GL from T5-T8, consistent with previous studies [13]. This may suggest the onset of fatigue accumulation and the recruitment of new MUs. Subsequently, as observed in Figure 3A, the EMG activity of GL remained unchanged during T9-T10, potentially due to signal loss caused by amplitude cancellation of muscle Motor Unit Action Potentials (MUAPs) prior to task failure [36].

In the current study, the RMS of the TA peaked at T8 and then significantly fell, while the RMS of the prime mover remained unchanged, as seen in Figure 2A,C. This parallels previous findings on antagonist muscles in the calf during isometric contraction fatigue experiments [13]. Some researchers [17,37] propose this as a spinal-level control mechanism that balances the activity between the agonist and antagonist muscles. In the absence of regulation by this mechanism, the torque generated by the antagonist muscle would increase with excitability and counteract the torque of the agonist muscle [14], potentially leading to task failure.

Therefore, according to our results, the activation level of TA continues to increase in the early-to-mid stages of the fatigue task, maintaining a similar trend as GL. However, prior to the end of the fatigue task (T9-T10), the RMS of TA decreases while that of GL remains constant. This could be due to a decrease in GL’s force-generating capability, with the cortex maintaining a constant co-activation level by inhibiting TA’s activity, thereby preserving movement stability.

### 4.2. EMG Frequency Domain Changes in the Agonist GL and Antagonist TA Muscles

The average and median frequencies of electromyography (EMG) are primarily influenced by the conduction velocity of Motor Unit Action Potentials (MUAPs) along muscle fibers and the degree of MUAPs clustering [15,38]. As fatigue increases, the conduction velocity of MUAPs along muscle fibers decreases, or when the MUAPs cluster more tightly, the median frequency of the EMG will drop. Our results reveal a continuous decline in the Median Frequency (MF) of the Gastrocnemius Lateralis (GL) throughout the fatigue process, consistent with previous findings on dynamic fatigue [32,39]. Based on the theory proposed by von Tscharner et al. [38], this might indicate an increase in the degree of clustering of GL’s MUAPs and a decrease in their conduction velocity with the onset of fatigue.

Our study shows a continuous decrease in the MF of the Tibialis Anterior (TA), with a significant drop occurring from T5 to T10. This aligns with the trend observed in the MF changes of antagonist muscles during elbow isometric fatigue tasks by Wang et al. [40]. Coupled with their findings on changes in the conduction velocity of antagonist muscles, they attributed this to peripheral fatigue caused by the accumulation of peripheral metabolites due to prolonged co-activation. Contrarily, a study [41] found no fatigue in the antagonist muscles after completing an isokinetic knee extension fatigue task, as there was no decline in torque and EMG amplitude. However, our results indicate a decrease in the MF of the antagonist muscles and a drop in RMS from T8 to T10, suggesting that TA, as an antagonist muscle, experienced peripheral fatigue. The effects of dynamic and static fatigue, as well as fatigue processes at different force levels, on antagonist muscles remain unclear and warrant further investigation.

Many studies [21,42,43,44] have pointed out that the power in the beta and gamma frequency bands of EMG is related to the synchronization activity between Motor Units (MUs). Simulated EMG study by von Tscharner et al. [38] also indicated that the higher the power in the part below 60 Hz, the stronger the synchronous activity of MUs. Our results show that during T1-T8, the power in the low-frequency range of both GL and TA increased, which is consistent with previous research [45,46]. This may suggest that during the early and middle parts of the fatigue task (T1–T8), neural drive to MUs becomes more synchronized to adapt to the effects of fatigue [45,47]. However, just before the end of the fatigue task (T10), the power in the low-frequency range of TA showed a significant decline. Few studies have reported on the changes in the power of different frequency bands of antagonist muscles throughout the fatigue task. Combined with the decline in RMS of the antagonist muscle, we speculate that this is due to the central nervous system reducing the activity of TA by inhibiting the synchronization of its MUs. This might serve as another piece of evidence for the differentiated control of antagonist muscles at the spinal level [14].

The power of EMG > 100 Hz might be related to the number of MUAPs. Our results show that the power in the high-frequency part of GL and TA EMG continuously increased during T1–T8, and began to decrease during T9-T10. Some research [48] suggests that the power of high-frequency components of EMG could be due to the rapid generation of MUAPs by fast motor units. The EMG model by von Tscharner et al. [38] also pointed out that the power in the high-frequency part of EMG is proportional to the number of MUAPs. The fatigue model by Potvin et al. [8] also predicts that the discharge rate of MUs continues to increase during fatigue, and suddenly decreases just before the task ends, which is consistent with our observations. This may indicate that the MU firing rate of TA and GL begins to decrease a short time before exhaustion (T9-T10), until task failure. Interestingly, the RMS of EMG did not show this change. A possible explanation is that when the discharge rate of MUs decreases and the number of MUAPs decreases close to task failure, the power of high-frequency EMG [38], which is proportional to the number of MUAPs, decreases. When the density of randomly distributed MUAPs is high, the cancellation effect will reduce the effective amplitude of EMG. When the density of MUAPs decreases, the cancellation effect also weakens, which might be reflected as unchanged EMG amplitude. Therefore, the power in the high-frequency range of EMG might indirectly reflect the discharge rate of MUs.

### 4.3. Coactivation of the Agonist GL and Antagonist TA Muscles

Coactivation, the phenomenon of antagonist and agonist muscle groups being activated simultaneously, is intended to maintain a specific level of neuromuscular function [49]. The phenomenon of coactivation is often explained by De Luca’s “common drive” theory [2]. This hypothesis suggests that when two muscles are involved in a specific task, the central nervous system can control the motor neuron pool of each muscle through a single input. This means that when one muscle contracts, the antagonist muscle will also contract simultaneously to coordinate and balance movement. On the contrary, some researchers believe that the central nervous system controls the agonist and antagonist muscles through different mechanisms: one that simultaneously activates the agonist and antagonist muscles, and another that activates the agonist while inhibiting the antagonist [13,14].

Our study results show that during fatigue, the coactivation level of TA and GL remains unchanged throughout the fatigue process. This is consistent with previous fatigue studies [11,13,50]. However, our results during T10 show that although the level of coactivation remains unchanged, the RMS of TA significantly decreases, and the power in the low-frequency range of TA significantly decreases as well, while there are no significant changes in the RMS and low-frequency power of GL. The “common drive” hypothesis has difficulty explaining this phenomenon; our results seem to support the view that the central nervous system controls the activities of agonist and antagonist muscles through multiple mechanisms.

### 4.4. EMG-EMG Coherence of the Agonist GL and Antagonist TA Muscles

The coherence in the alpha band originates from the subcortex and may reflect the ascending or feedback interactions in neuromuscular control [5]. A study [11] found that fatigue in healthy adults resulted in a significant increase in coherence in the alpha band during isometric fatigue tasks at the elbow. However, in contrast, our results show that there were no significant changes in the coherence of the alpha band during the fatigue process. This might suggest that the ascending or feedback interaction is stable in dynamic fatigue. Another possibility is that the activity of Renshaw cells in the spinal cord could influence the coherence in the alpha band, leading to inconsistent observations of coherence in the alpha band in many studies [5].

Evidence suggests that the coherence in the beta and gamma bands is mainly controlled by the motor cortex and can reflect the coupled action of the cortex and muscles [51,52]. Many previous studies have explored the corticomuscular and intermuscular coherence in the beta band [44,53,54,55]. Baker [55] observed in monkeys that the coherence between the cortex and muscles in the beta band significantly increased when completing a posture maintenance task. The same results were found in humans, where the intermuscular coherence in the beta band among FDI [44], index finger flexor [10], knee extensor [52], and elbow antagonist [11] increased during muscle fatigue caused by sustained isometric maximal or dynamic movements. The coherence in the gamma band is often observed in dynamic tasks. Previous studies have observed significant EEG-EMG coherence in the gamma band during maximum voluntary contraction [51], and the coherence in the gamma band is related to the level of force [56].

Our results show that the EMG-EMG coherence in the beta and gamma bands gradually increases, with significant changes appearing in the middle of the fatigue task (T5-T6) and reaching a peak at T8-T9, but without significant changes. Similar results have been found in previous studies [7]. High coherence between EMG-EMG might indicate that the two muscles share neural drive [54], and fatigue has been observed to enhance the EMG-EMG coherence between agonist and antagonist muscles. Based on this, we speculate that in the middle of our fatigue task (T5–T6), fatigue leads to a decline in muscle function, and the central nervous system needs to enhance the driving effect on the agonist and antagonist muscles to compensate for the impact of fatigue.

Contrary to our results, Wang et al. [19] found that during a 30% MVC isometric fatigue task, there was no significant change in the intermuscular coherence in the beta and gamma bands between the agonist and antagonist muscles of the elbow before and after fatigue. This may be related to the choice of muscles; the study chose proximal elbow muscles, while other research suggests that distal muscles have stronger cortical neuron connections. This could lead to different levels of coupling between distal and proximal muscles and the central nervous system [10]. Another possible reason is the difference in coherence analysis methods; the study used the Fourier method to study coherence, which might affect the reliability of the results [23].

Interestingly, the EMG-EMG coherence in the beta band of the GL and TA showed significant changes at T6, while the EMG-EMG coherence in the gamma band showed significant changes at T5. These changes do not coincide with the time when the RMS of the antagonist or agonist muscles reached their maximum. EMG-EMG coherence is often considered a tool to quantify neuromuscular coupling [1]. The continuous increase in EMG-EMG coherence from T1 to T6, along with the increase in RMS of GL and TA, might suggest that as fatigue occurs, neuromuscular coupling continues to strengthen. This effect is manifested at the muscle activity level as an increase in EMG amplitude. However, after T6, the increase in EMG-EMG coherence slowed down significantly, and no significant changes appeared. But the RMS of GL and TA continues to increase until T8. The reason for this change is not clear. A study on synergistic muscles found that personalized control of muscles leads to a decrease in beta coherence between muscles [57]. This might indicate that at T6, the central nervous system began to differentiate control of the antagonist muscles, and this control appeared before the strongest activity of the antagonist muscles. However, we did not collect direct indicators of central fatigue, so this connection is speculative. Future research can provide more evidence and explanations for this issue.

Indeed, since coherence can reflect the communication between the cortex and muscles, it is often used to monitor the recovery status of neuromuscular function [1,58]. Moderate fatigue can enhance athletic performance [19], but excessive fatigue can reduce performance and increase the risk of injury [59]. Therefore, detecting when fatigue occurs and the extent of fatigue is of great significance. Our study identified a potentially significant transition point at 60% of the fatigue task, where neuromuscular control strategies appear to shift. However, current research on this specific time point is limited, making it challenging to provide concrete training recommendations. We suggest that future research focus on this 60% time point, studying the effects of different training interventions. This could lead to more effective, individualized training protocols that optimize performance while minimizing injury risk. In the meantime, trainers could consider monitoring EMG-EMG coherence and EMG changes of antagonist muscles during training sessions to gain insights into fatigue development and neuromuscular control strategies.

### 4.5. Limitation

There are several limitations in this study. Firstly, our EMG collector has a built-in 20–450 Hz LTI filter, and the frequency bands (alpha, beta) we are interested in overlap with this frequency segment. According to the research of Chen et al. [23], theoretically, preprocessing with an LTI filter will not improve or affect coherence estimation. The factors that affect coherence are mainly the calculation methods. Using the Welch method to estimate spectral density for coherence analysis may lead to increased variance or spectral leakage in results, thereby affecting the outcome. In our study, we adopted wavelet coherence analysis. Secondly, this study did not collect indicators that can directly reflect central fatigue, so we cannot directly discuss the state of central fatigue. Future research can measure corticospinal excitability to discuss the effect of central fatigue on neuromuscular control. Thirdly, the sample in this study is limited to healthy male college students aged 18–25 without investigating their physical activity levels. Future research could include participants of different ages, genders, and fitness levels (ranging from sedentary to highly active) to understand how population characteristics affect neuromuscular control during fatigue.

## 5. Conclusions

Our research results reveal changes in neuromuscular control during the dynamic fatigue process. In the first 60% of the fatigue task, the beta and gamma band EMG-EMG coherence of the calf’s agonist and antagonist muscles continuously increase, and this increase presents a synchronous trend with the changes in muscle EMG amplitude. This finding may suggest that during the first 60% of the task, the central nervous system maintains the stability of the exercise level by continuously enhancing the driving effect on the muscles. Interestingly, after the task progresses to 60%, the ENG-EMG coherence remains stable, while the EMG amplitude of the GL and TA continues to increase. This might be because the central nervous system maintains the level of co-activation by differentiating the control of the antagonist muscles through mechanisms other than co-driving, and this differentiated control appears before muscle activity. This implies that the central nervous system adopts more complex and refined strategies to regulate muscle activity under fatigue. Based on our research results, we believe that monitoring EMG-EMG coherence and the EMG changes of antagonist muscles may be of significant importance in the process of athletic training. This monitoring method may provide a basis for the evaluation and monitoring of training effects, thereby helping trainers better understand the development of muscle fatigue and adopt corresponding training adjustment measures.

## Data Availability

The data presented in this study are available on request from the corresponding author. The data are not publicly available due to privacy and ethical restrictions, as they contain information that could compromise the privacy of research participants.
